# Defect-Induced π-Magnetism into Non-Benzenoid Nanographenes

**DOI:** 10.3390/nano12020224

**Published:** 2022-01-11

**Authors:** Kalyan Biswas, Lin Yang, Ji Ma, Ana Sánchez-Grande, Qifan Chen, Koen Lauwaet, José M. Gallego, Rodolfo Miranda, David Écija, Pavel Jelínek, Xinliang Feng, José I. Urgel

**Affiliations:** 1 IMDEA Nanoscience, C/Faraday 9, Campus de Cantoblanco, 28049 Madrid, Spain; kalyan.biswas@imdea.org (K.B.); ana.sanchez@imdea.org (A.S.-G.); koen.lauwaet@imdea.org (K.L.); rodolfo.miranda@imdea.org (R.M.); 2 Center for Advancing Electronics, Faculty of Chemistry and Food Chemistry, Technical University of Dresden, 01062 Dresden, Germany; Lin.Yang1@tu-dresden.de (L.Y.); Xinliang.Feng@tu-dresden.de (X.F.); 3 Institute of Physics of the Czech Academy of Science, CZ-16253 Praha, Czech Republic; chenq@fzu.cz; 4 Instituto de Ciencia de Materiales de Madrid, CSIC, Cantoblanco, 28049 Madrid, Spain; josemaria.gallego@imdea.org; 5 Departamento de Física de la Materia Condensada, Universidad Autónoma de Madrid, 28049 Madrid, Spain; 6 Regional Centre of Advanced Technologies and Materials, Palacký University Olomouc, CZ-77146 Olomouc, Czech Republic

**Keywords:** on-surface synthesis, nanomagnetism, polycyclic aromatic hydrocarbons, nanographenes, open-shell character, STM, nc-AFM

## Abstract

The synthesis of nanographenes (NGs) with open-shell ground states have recently attained increasing attention in view of their interesting physicochemical properties and great prospects in manifold applications as suitable materials within the rising field of carbon-based magnetism. A potential route to induce magnetism in NGs is the introduction of structural defects, for instance non-benzenoid rings, in their honeycomb lattice. Here, we report the on-surface synthesis of three open-shell non-benzenoid NGs (**A_1_**, **A_2_** and **A_3_**) on the Au(111) surface. **A_1_** and **A_2_** contain two five- and one seven-membered rings within their benzenoid backbone, while **A_3_** incorporates one five-membered ring. Their structures and electronic properties have been investigated by means of scanning tunneling microscopy, noncontact atomic force microscopy and scanning tunneling spectroscopy complemented with theoretical calculations. Our results provide access to open-shell NGs with a combination of non-benzenoid topologies previously precluded by conventional synthetic procedures.

## 1. Introduction

The physicochemical properties of well-defined compounds that consist of fused conjugated aromatic rings, frequently referred as to polycyclic aromatic hydrocarbons or NGs [1], have been under the spotlight over the last years due to their potential in great number of technological applications [2,3,4]. Tuning such properties is feasible by modifying some of their structural characteristics as: (i) size, (ii) edge topology [5,6] or (iii) by introducing structural defects in the honeycomb lattice [7,8]. While the majority of NGs, known to be model compounds in organic chemistry, accommodates π-electrons in the bonding orbitals conferring them a closed-shell singlet ground state; compounds comprising unpaired or partially unpaired electrons within the molecular backbone, i.e., with an open-shell ground state, display unique electronic properties capable of carrying magnetism and conductivity functionalities [9,10,11,12]. However, their high reactivity usually makes conventional solution-mediated synthesis of open-shell NGs a great challenge, limiting the number of available compounds.

The synthesis of novel reactive compounds confined on a metallic surface under ultrahigh vacuum (UHV) conditions has emerged as a compelling alternative synthetic toolbox toward the design of open-shell NGs [13]. For instance, the nature of the electronic ground state of long pursued members of the acene [14,15,16,17,18,19] and triangulene [20,21] families, widely discussed in theoretical studies, have only recently been untangled. Contemporarily, a successful strategy toward the on-surface formation of open-shell NGs, one-dimensional polymers and graphene nanoribbons (GNRs) is the surface-assisted oxidative ring closure between a methyl group and the neighboring aryl moiety of a properly predesigned molecular precursor, which occurs after thermal activation [22,23,24,25,26,27,28,29,30,31,32,33,34,35,36,37]. Such synthetic approach, though often successful, presents some limitations related to the cleavage of methyl groups prior to cyclization, which may lead to the formation of topological defects inducing an open-shell ground state to NGs [30,37].

**Scheme 1:**
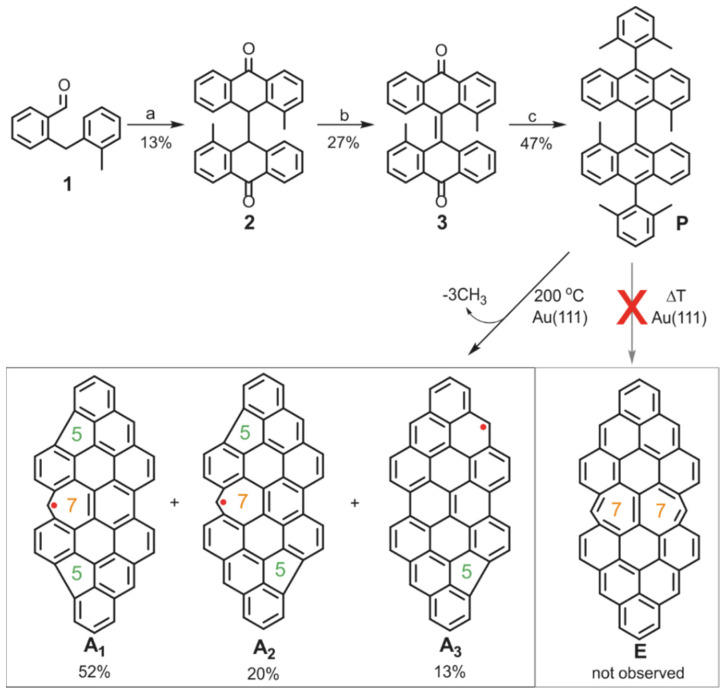
Conceptual routes toward the formation of open-shell non-benzenoid NGs on Au(111). Reagents and conditions: (**a**) K_2_S_2_O_8_, tetraethylammonium bromide, 1,2-dichloroethane, 120 °C, 36 h. (**b**) (i) KOH, EtOH, reflux, 0.5 h; (ii) K_2_S_2_O_8_, H_2_O, rt., 2 h. (**c**) (i) 2,6-dimethylphenylmagnesium bromide, THF, 0 °C-rt., 24 h; (ii) NaI, NaH_2_PO_2_·H_2_O, CH_3_COOH, reflux, 2 h.

In this article, we report the synthesis of three open-shell non-benzenoid NGs (**A_1_**, **A_2_** and **A_3_**) with a total spin (S) = 1/2 in their atomic lattice, resulting from the on-surface reactions of the 10,10′-bis(2,6-dimethylphenyl)-1,1′-dimethyl-9,9′-bianthracene precursor (P) on Au(111) in a UHV environment (Scheme 1). Our attempts to synthesize the expected heptalene-embedded NG (**E**) on the gold surface were ineffective due to the propensity of methyl groups to cleavage prior to oxidative ring closure. The chemical structure of the three NGs is clearly determined by scanning tunneling microscopy (STM) and noncontact atomic force microscopy (nc-AFM). Moreover, their electronic properties are studied by scanning tunneling spectroscopy (STS) and complemented by density functional theory (DFT) calculations, which indicates the presence of an unpaired spin detected as a Kondo resonance.

## 2. Experimental Details

Experiments were performed in a custom-designed ultra-high vacuum system (base pressure below 4 × 10^−10^ mbar) hosting a commercial low-temperature microscope with STM/AFM capabilities from Scienta Omicron and located at IMDEA Nanoscience (Madrid, Spain).

The Au(111) substrate was prepared by repeated cycles of Ar^+^ sputtering (E = 1.5 keV) and subsequent annealing at 740 K for 10 min. All STM images shown were taken in constant-current mode, unless otherwise noted, with electrochemically etched tungsten tips, at a sample temperature of 4.3 K (LakeShore, Carson, CA, USA). Scanning parameters are specified in each figure caption. The molecular precursor was thermally deposited (Kentax TCE-BSC, Seelze, Germany) onto the clean Au(111) surface held at room temperature with a typical deposition rate of 0.5 Å/min (sublimation temperature of 170 °C), controlled by a quartz micro balance (LewVac, Burgess hill, United Kingdom). After deposition of P, the sample was post-annealed at 200 °C for 10 min to induce the cyclodehydrogenation reaction.

Non-contact AFM measurements were performed with a tungsten tip attached to a Qplus tuning fork sensor (Omicron, Taunusstein, Germany) [38]. The tip was functionalized a posteriori by the controlled adsorption of a single CO molecule at the tip apex from a previously CO-dosed surface [39]. The functionalized tip enables the imaging of the intramolecular structure of organic molecules [40]. The sensor was driven at its resonance frequency (~26 kHz for Qplus) with a constant amplitude of ~80 pm. The shift in the resonance frequency of the sensor (with the attached CO-functionalized tip) was recorded in a constant-height mode (Omicron Matrix electronics and MFLi PLL by Zurich Instruments (Zurich, Switzerland) for Omicron). The STM and nc-AFM images were analyzed using WSxM 5.0 (Madrid, Spain) [41].

## 3. Results and Discussion

### 3.1. On-Surface Synthesis of Non-Benzenoid Nanographenes

**Figure 1:**
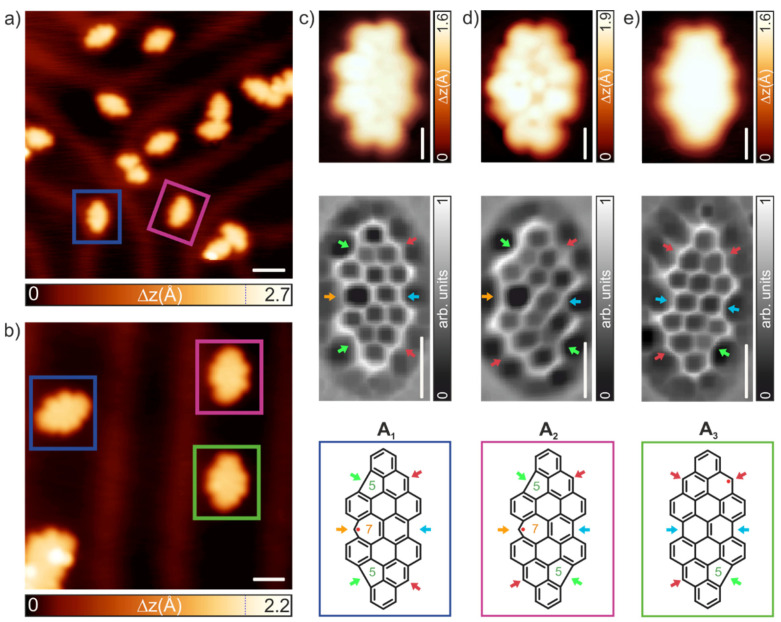
Synthesis and structural characterization of non-benzenoid NGs on Au(111). (**a**,**b**) Overview STM images of the Au(111) surface after sublimation of **P** and subsequent annealing at 200 °C. Scanning parameters: (**a**) *V*_b_ = −1.5 V, *I*_t_ = 10 pA and (**b**) *V*_b_ = −1.5 V, *I*_t_ = 100 pA. (**a**,**b**) Scale bars = 2 and 1 nm, respectively. (**c**–**e**) High-resolution STM images (top row) and constant-height frequency-shift nc-AFM (middle row) acquired with a CO-functionalized tip, together with the corresponding non-benzenoid non-Kekulé chemical structure (bottom row) of **A_1_** (**c**), **A_2_** (**d**) and **A_3_** (**e**). The colored rectangles of the distinct NGs correspond to the colored ones in (**a**,**b**) (blue (**A_1_**), violet (**A_2_**), and green (**A_3_**)). The colored arrows highlight the formation of new rings as described in the main text. STM parameters: *V*_b_ = 5 mV, *I*_t_ = 30 pA, all scale bars = 0.5 nm. Nc-AFM parameters: Z offset = 150 pm above the STM set point (5 mV, 50 pA), scale bars = 1 nm.

Since the synthesis of **E** through conventional solution synthesis methods was unsuccessful, we directed our attention to the on-surface synthesis approach. Thus, a first step toward the formation of a heptalene-embedded non-benzenoid nanographene involves the synthesis of the molecular precursor **P**, which was prepared by solution chemistry. As shown in Scheme 1, compound **2** was firstly obtained by the oxidative acylation reaction and homo-coupling of 2-(2-methylbenzyl)benzaldehyde **1**, which was then enolized and dehydrogenated to yield the bisanthrone derivative **3**. After that, derivative **3** was treated with 2,6-dimethylphenylmagnesium bromide, followed by reduction to afford 10,10′-bis(2,6-dimethylphenyl)-1,1′-dimethyl-9,9′-bianthracene precursor (**P**). The six methyl groups from **P** are expected to undergo surface-catalyzed oxidative ring closure, and the final compound **E** is expected to comprise two triangulene subunits connected by two heptagons. With this aim, a low coverage of **P** (0.1 ML) was deposited onto an Au(111) surface held at room temperature. Subsequent annealing of the sample at 200 °C affords the formation of distinct non-benzenoid NGs coexisting with some fused nanostructures, as observed in the STM images shown in Figure 1a,b. The formation of such NGs is attributed to the oxidative ring closure of the majority of the methyl groups, together with the dissociation of several of them per NG prior to cyclization. Such removal of methyl moieties was previously reported in the synthesis of several NGs [30,37,42,43,44,45] and GNRs [22,46] and is concomitant to the annealing step at temperatures where the oxidative ring closure is expected to occur on Au(111), therefore being not possible to achieve the synthesis of **E**.

In order to obtain further structural information of the formed NGs, constant-height frequency-shift nc-AFM measurements acquired using a CO-terminated tip were performed [40,47]. The majority (≈85%) of the formed NGs (A_1_–A_3_) are shown in Figure 1c–e. All of them feature a planar conformation on the surface. The images depicting **A_1_** (Figure 1c) and **A_2_** (Figure 1d) allow us to discern the formation of two five- and one six-membered rings, attributed to the loss of three methyls (green and blue arrows, respectively), together with the expected formation of one seven-membered ring (orange arrows) and two six-membered rings via oxidative ring closure (red arrows). The main structural difference between both NGs is the location of one of the formed five-membered rings, depending on which methyl detached prior to oxidative ring closure from the (dimethyl) phenyl subunits. Similarly, **A_3_** (Figure 1e) arises from the loss of three methyls, giving rise to the formation of one five- and two six-membered rings (green and blue arrows, respectively)_,_ together with the expected formation of three six-membered rings (red arrows). In addition, a minority of benzenoid NGs [36], together with methyl migration and some fused species were observed on the Au(111) surface (see Figure S1 for their structural characterization). Therefore, **A_1_**–**A_3_** incorporate odd-membered rings at different positions of their atomic lattice in which a non-Kekulé structure is expected with a total spin S = ½, as shown in the chemical sketches in Figure 1c–e [10,29,48,49,50].

### 3.2. Electronic and Magnetic Characterization of Non-Benzenoid Nanographenes

**Figure 2:**
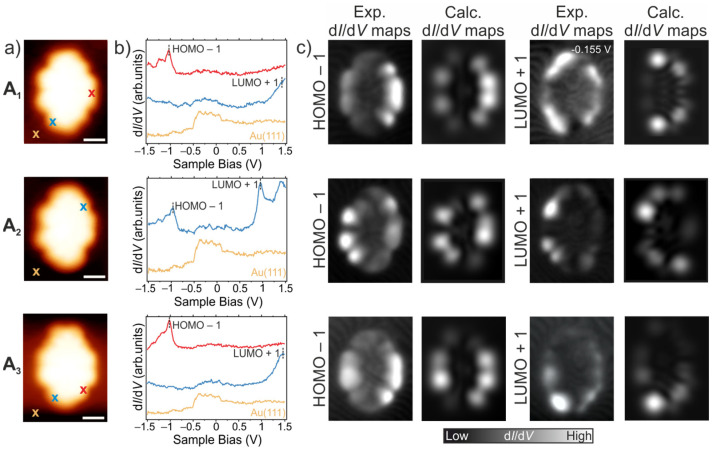
Electronic characterization of **A_1_**–**A_3_** on Au(111). (**a**) High-resolution STM images acquired with a CO-functionalized tip displaying the NGs marked with colored squares in (**a**). Scanning parameters: *V*_b_ = −1.5 V, *I*_t_ = 100 pA, all scale bars = 0.5 nm. (**b**) d*I*/d*V* spectra of **A_1_**–**A_3_** acquired at the positions indicated by the blue and red crosses in (**b**). Reference spectra taken on the bare Au(111) surface is depicted in orange and the acquisition positions marked with an orange cross. Open feedback parameters for d*I*/d*V* spectra: *V*_b_ = −1.5 V, *I*_t_ = 250 pA, V_rms_ = 10 mV. (**c**) Constant-current differential conductance (d*I*/d*V*) maps and corresponding DFT-calculated maps acquired with a CO-tip of the free-standing NGs (tip-sample height = 5 Å) at the energetic positions corresponding to the HOMO − 1 (left) and the LUMO + 1 (right). Scanning parameters: **A_1_**; *V*_b_ = −980 mV, *I*_t_ = 300 pA (HOMO − 1), *V*_b_ = 1400 mV, *I*_t_ = 300 pA (LUMO + 1). **A**_2_; *V*_b_ = −980 mV, *I*_t_ = 250 pA (HOMO − 1), *V*_b_ = 880 mV, *I*_t_ = 250 pA (LUMO + 1). **A_3_**; *V*_b_ = −980 mV, *I*_t_ = 300 pA (HOMO − 1), *V*_b_ = 1400 mV, *I*_t_ = 300 pA (LUMO + 1).

Next, we have inspected the electronic structure of the different non-benzenoid NGs (Figure 2a–c) via STS. The long-range differential conductance d*I*/d*V* spectra acquired on **A_1_**–**A_3_** NGs show prominent resonances in the local density of states at around −0.9 V and +1.4 V for **A_1_**, −0.9 V and +1.0 V for **A_2_**, −1.0 V and +1.4 V for **A_3_** (Figure 2b). By acquiring d*I*/d*V* maps at those specific bias voltages and comparing them to the calculated d*I*/d*V* maps [47,51], such resonances are assigned to HOMO − 1 (HOMO = highest occupied molecular orbital) and LUMO + 1 (LUMO = lowest unoccupied molecular orbital), respectively, as illustrated in Figure 2c. In addition, the trend in the energy gap between HOMO − 1 and LUMO + 1 for **A_1_**–**A_3_** is in agreement with the one displayed by DFT calculations [52,53] of the free-standing NGs (see Figure S2), which altogether corroborates our rationalization of the electronic structure of the non-benzenoid species under study.

**Figure 3:**
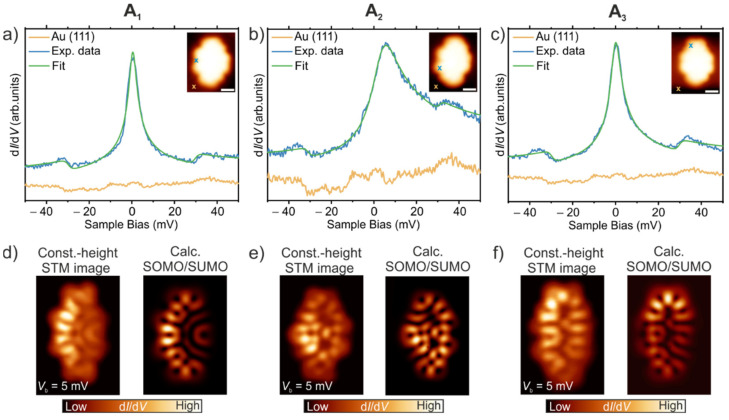
Open-shell character of **A_1_**–**A_3_**. (**a**–**c**) Short-range d*I*/d*V* spectra acquired at specific locations of **A_1_**–**A_3_**. The blue curves display the experimental data, and the green curves the corresponding Frota function fit. Orange curves depict the reference d*I*/d*V* spectra acquired on Au(111). Open feedback parameters: *V*_b_ = −50 mV, *I*_t_ = 450 pA, V_rms_ = 2 mV. (**d**–**f**) Constant-height STM images acquired at low bias voltages (*V*_b_ = 5 mV, *I*_t_ = 30 pA) and their comparison with the corresponding DFT-calculated maps of the SOMO/SUMO.

The open-shell non-Kekulé structures depicted in Figure 1c–e suggest that **A_1_**–**A_3_** should present an unpaired spin (S = ½) per NG, having thus an open-shell ground state by definition [11]. Systems presenting an unpaired spin on a metallic substrate are typically expected to exhibit a Kondo resonance [54]. In order to demonstrate the singlet open-shell character of **A_1_**–**A_3_**, we have recorded d*I*/d*V* spectra at low bias voltages to observe any magnetic fingerprint. Interestingly, Figure 3a,c show pronounced low-bias peaks centered around the Fermi energy which are assigned to Kondo resonances, and can be nicely fitted by a Frota function, as expected for the Kondo phenomenon [55]. The determined half width half maxima (HWHM) are 4.4 mV and 5.7 mV, indicating a Kondo temperature of 23 K and 47 K for **A_1_** and **A_3_**, respectively. Figure 3b shows a spectrum in the same bias range measured on **A_2_**. Again, a feature at low bias voltages is observed, but it is noticeably broader (HWHM of 15.4 mV), and has a slight offset toward positive bias values, centered at 3.3 mV. We tentatively attribute this feature to the orbital holding the unpaired spin, which is now above the Fermi level due to charge transfer with the underlying substrate. While long-range d*I*/d*V* spectroscopy on **A_1_**–**A_3_** provides clear evidence of the non-frontier states (HOMO − 1 and LUMO + 1), no explicit signatures of the frontier states (SOMOs = singly occupied molecular orbitals and SUMOs = singly unoccupied molecular orbitals) were observed [33,56]. However, constant-height STM images recorded close to the Fermi level resemble the shape of the calculated SOMO and SUMO (see Figure 3d–f), which corroborates the open-shell character of the studied NGs.

## 4. Conclusions

In conclusion, we have demonstrated the on-surface synthesis of well-defined open-shell non-benzenoid NGs (**A_1_**–**A_3_**) on Au(111), via on-surface oxidative ring closure and methyl detachment of the parent precursor **P** upon annealing at 200 °C; and their structures have been clearly elucidated by STM and nc-AFM. Two of such NGs present one seven- and two five-membered rings (**A_1_** and **A_2_**), while the last species features one five-membered ring (**A_3_**). Importantly, the presence of such non-benzenoid rings render **A_1_**–**A_3_** as non-Kekulé structures which are expected to host an unpaired electron (S = ½) per NG. STS studies, together with theoretical calculations, confirm the existence of such unpaired electron in two of the three species (**A_1_** and **A_3_**) through the measurement of a Kondo resonance on the NGs. **A_2_** on the other hand, also presents a feature above the Fermi level, but in this case such resonance is attributed to the partial filling of the SOMO orbital, due to charge transfer with the underlying surface. The synthetized NGs can serve as model structures that help to understand the introduction of odd-member rings in the honeycomb lattice of graphene nanostructures, paving avenues to engineer novel non-benzenoid NGs on surfaces of interest in molecular electronics and magnetism.

## Data Availability

The data presented in this study are available on request from the corresponding author.

## Supplementary Materials

The following supporting information can be downloaded at: https://www.mdpi.com/article/10.3390/nano12020224/s1, Figure S1: Constant-height frequency shift nc-AFM images of the minority NGs formed on the Au(111) surface. Figure S2: Scheme of calculated PDOS of the different open-shell nonbenzenoid NGs labeling the position of the frontier orbitals SOMO, SUMO, HOMO and LUMO.
