# Supplementary material for: Defect-Induced π-Magnetism into Non-Benzenoid Nanographenes
Source: Nanomaterials (Basel). 2022 Jan 11;12(2):224. doi: 10.3390/nano12020224 (PMC8780648; doi:10.3390/nano12020224)
Supplement: Supplementary file 1 [file nanomaterials-12-00224-s001.zip › nanomaterials-1536096-supplementary.pdf]

Supplemental materials for

Defect-Induced π-Magnetism into Non-Benzenoid Nanographenes

1. Solution synthesis general methods and materials

All the reagents were obtained from Sigma Aldrich (San Luis, MO, USA), TCI (Tokyo, Japan) or Strem (Newburyport, MA, USA). All these chemicals were used as received without further purification. All reactions dealing with air- or moisture-sensitive compounds were carried out in a dry reaction vessel under Ar atmosphere.

Thin layer chromatography (TLC) was performed on silica-coated aluminium sheets with a fluorescence indicator (TLC silica gel 60 F254, purchased from Merck KgaA, Darmstadt, Germany). Column chromatography was performed on silica (SiO_2, particle size 0.063-0.200 mm, purchased from VWR, Radnor, PA, USA).

NMR spectra were recorded on a Bruker (Billerica, MA, USA) AV-II 300 spectrometer operating at 300 MHz for ^1H and at 75 MHz for ^{13}C at room temperature. $CDCl_3$ ($\delta(^1H)$ = 7.26 ppm, $\delta(^{13}C)$ = 77.0 ppm), CD_2Cl_2 ($\delta(^1H)$ = 5.33 ppm, $\delta(^{13}C)$ = 53.7 ppm) was used as solvents and as internal chemical shift reference. Chemical shifts (δ) are reported in ppm. The following abbreviations are used to describe peak patterns as appropriate: s = singlet, d = doublet, t = triplet, q = quartet, and m = multiplet.

The mass spectrometry analysis was performed on a Bruker Autoflex Speed MALDI TOF MS (Bruker Daltonics, Bremen, Germany) using *trans*-2-[3-(4-*tert*-butylphenyl)-2-methyl-2-propenylidene]malononitrile (DCTB) as matrix.

2. Synthetic procedure toward precursor P

2.1. Synthesis of P

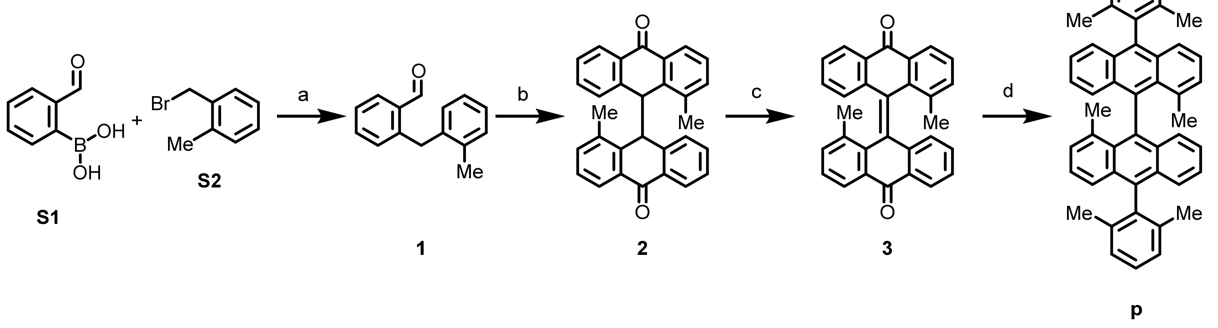

Scheme S1. Synthetic route of ***P***. (**a**) $Pd(PPh_3)_4$, Na_2CO_3, toluene/ H_2O, 100 °C, 12 h, 77%. (**b**) $K_2S_2O_8$, tetraethylammonium bromide, 1,2-dichloroethane, 120 °C, 36 h, 13%. (**c**) i) KOH, EtOH, reflux, 0.5 h; ii) $K_2S_2O_8$, H_2O, rt., 2 h, 27%. (**d**) i) RMgBr, THF, 0 °C - rt., 24 h; ii) NaI, $NaH_2PO_2{\bullet}H_2O$, CH_3COOH, reflux, 2 h, 47%.

2.2 Synthesis of 2-(2-methylbenzyl)benzaldehyde (1)

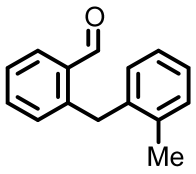

1

A flask was charged with commercially available (2-formylphenyl)boronic acid (**S1**, 1.8 g, 12 mmol) and Na_2CO_3 (2.1 g, 20 mmol) along with 30 mL of toluene and 10 mL water. The mixture was degassed by Ar bubbling and then $Pd(PPh_3)_4$ (577.5 mg, 0.5 mmol) and 1-(bromomethyl)-2-methylbenzene (**S2**, 1.34 mL, 10 mmol) were added. The solution was further degassed by Ar bubbling for another 5 min. Then the reaction was stirred at 100 ℃ under Ar for 12 h. Afterwards, the reaction was quenched with water (10 mL). The mixture was extracted with CH_2Cl_2 (20 mL×3), dried over $MgSO_4$, and evaporated. The crude product was purified by column chromatography (*iso*-hexane/DCM=3:1) to give the compound **1** as a colorless oil (1.62 g, 77%). ^1H NMR (300 MHz, $CDCl_3$) δ 10.08 (s, 1H), 7.78 – 7.68 (m, 1H), 7.37 – 7.22 (m, 2H), 7.08 – 6.87 (m, 4H), 6.73 (d, J = 7.2 Hz, 1H), 4.28 (s, 2H), 2.12 (s, 3H). The ^1H NMR spectrum is consistent with the literature reported[1].

2.3 Synthesis of 1,1′-dimethyl-[9,9′-bianthracene]-10,10′(9H,9′H)-dione (2)

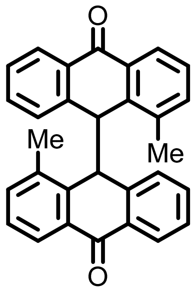

2

To a 50 mL thick pressure tube was added **1** (1.26 g, 6 mmol), $K_2S_2O_8$ (3.25 g, 2.0 equiv) and tetraethylammonium bromide (204.2 mg, 10 mol). Then, the tube was purged with Ar for three times, followed by addition of degassed 1,2-dichloroethane (30 mL). The formed mixture was stirred at 120 ºC under Ar for 36 h. The solution was then cooled to rt, and the solvent was removed under vacuum directly. The crude product was purified by column chromatography on silica gel (*iso*-hexane /ethyl acetate =20:1) to afford 322 mg (13 %) of **2,** there is still some starting material after the reaction. The ^1H NMR spectrum is much more complex than expected from the chemical structure due to the isomers. ^1H NMR (300 MHz, CD_2Cl_2) δ 7.73 – 7.63 (m, 4H), 7.40 – 7.32 (m, 2H), 7.28 – 7.18 (m, 4H), 7.02 – 6.92 (m, 2H), 5.94 – 5.84 (m, 2H), 4.81 – 4.59 (m, 2H), 2.91 – 1.84 (m, 6H). ^{13}C NMR (75 MHz, CD_2Cl_2) δ 182.72, 182.55, 140.26, 135.57, 135.38, 135.04, 134.74, 134.45, 134.28, 133.95, 131.83, 130.60, 130.21, 127.81, 127.66, 127.47, 127.34, 125.74, 125.50, 124.42, 124.33, 47.63, 19.58.

2.4 Synthesis of 1,1′-dimethyl-10H,10′H-[9,9′-bianthracenylidene]-10,10′-dione (3)

In a 100 mL flask, **2** (123.7 mg, 0.3 mmol) was added to a solution of KOH (673.2 mg, 12 mmol) in EtOH (50 mL) under argon atmosphere, and the mixture was refluxed for 0.5 h. The dark red solution was filtered into a well stirred solution of $K_2S_2O_8$ in water (50 mL). After 2 h, the greenish-yellow mixture was collected by filtration, washed with water, and dried to give the crude compound. The mixture was subjected to chromatography on silica gel (*iso*-hexane /ethyl acetate =5:1) to get **3** as a yellowish solid (33.3 mg, 27%). The ^{1}H NMR spectrum is much more complex than expected from the chemical structure due to the isomers. ^{1}H NMR (300 MHz, $CDCl_3$) 8.22– 8.18 (m, 6H), 7.75 – 7.67 (m, 4H), 7.61 – 7.51 (m, 4H), 2.81 (s, 6H). The poor solubility of this compound prohibited its ^{13}C NMR characterization.

2.5 Synthesis of 10,10′-bis(2,6-dimethylphenyl)-1,1′-dimethyl-9,9′-bianthracene (P)

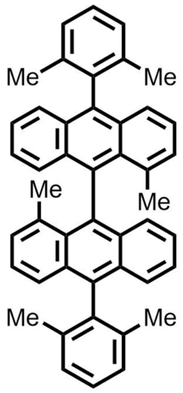

P

A solution of 2,6-dimethylphenylmagnesium bromide in THF (1 M, 0.5 mL) was added to the solution of **3** (20.6 mg, 0.05 mmol) in THF(1.5 mL) at 0 ºC under argon atmosphere. The reaction mixture was allowed to warm to room temperature and stirred for 24 h. After quenched with glacial acetic acid (0.5 mL), the solvents were removed under vacuum. Sodium iodide (75 mg, 0.5 mmol), sodium hypophosphite monohydrate (106 mg, 1 mmol) and glacial acetic acid (2 mL) were added to the residue obtained. The mixture was refluxed for 2 h. After cooled, the reaction was quenched with water (5 mL). The mixture was extracted with CH_2Cl_2 (10 mL×3), the organic layer was washed with water and saturated aqueous $NaHCO_3$, dried over anhydrous $MgSO_4$ and evaporated. The residue was purified with column chromatography on silica gel (*iso*-hexane/CH_2Cl_2=10/1) to give **P** as a yellow solid (13.8 mg, 47%). The ^{1}H NMR spectrum is much more complex than expected from the chemical structure due to the steric isomers. ^{1}H NMR (300 MHz, CD_2Cl_2) 7.61– 7.46 (m, 4H), 7.44 – 7.34 (m, 6H), 7.31 – 7.19 (m, 5H), 7.15 – 7.08 (m, 5H), 2.01 (s, 6H), 1.85 (s, 6H), 1.50 (s, 6H). ^{13}C NMR (CD_2Cl_2, 75 MHz): δ = 139.05, 138.07, 137.94, 137.86, 137.43, 136.79, 136.51, 133.54, 132.86, 132.61, 130.94, 129.80, 129.00, 128.14, 128.05, 128.02, 128.01, 127.99, 127.96, 127.71, 126.55, 126.20, 126.11, 126.06, 126.01, 125.64, 24.7, 20.3, 20.0 ppm. HR-MS (MALDI-TOF, m/z): calculated for [$C_{46}H_{38}$] $[M]^+$, 590.2968; found, 590.3134, error = + 2.81 ppm.

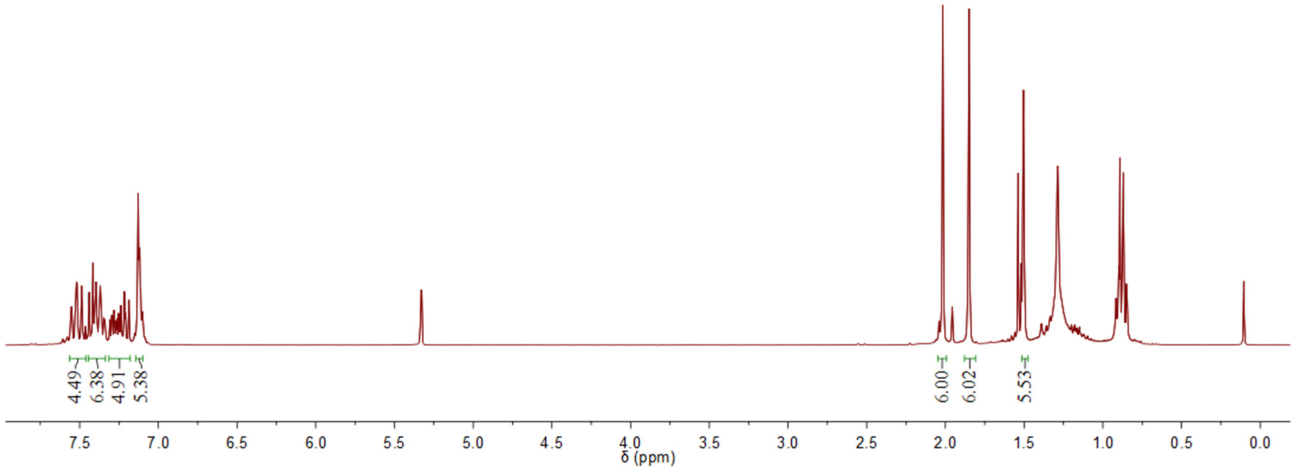

H NMR spectrum of **P** (solvent: CD_2Cl_2).

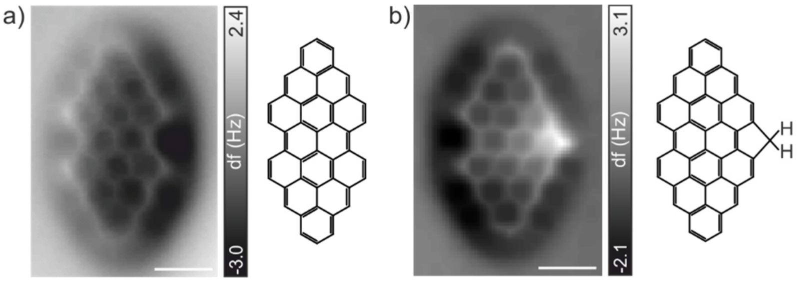

Figure S1. Constant-height frequency shift nc-AFM images of the minority NGs formed on the Au(111) surface. (**a**) High-resolution nc-AFM image acquired with a CO-functionalized tip and corresponding chemical sketch of a planar extended triangulene comprising a benzenoid lattice. (**b**) High-resolution nc-AFM image and corresponding chemical sketch of an extended triangulene with an extra carbon bond, tentatively attributed to a methyl migration from **P**, forming an extra hydrogenated five-membered ring. Scanning parameters: Z offset = 130 pm above the STM set point: 5 mV, 50 pA. Scale bars = 0.5 nm.

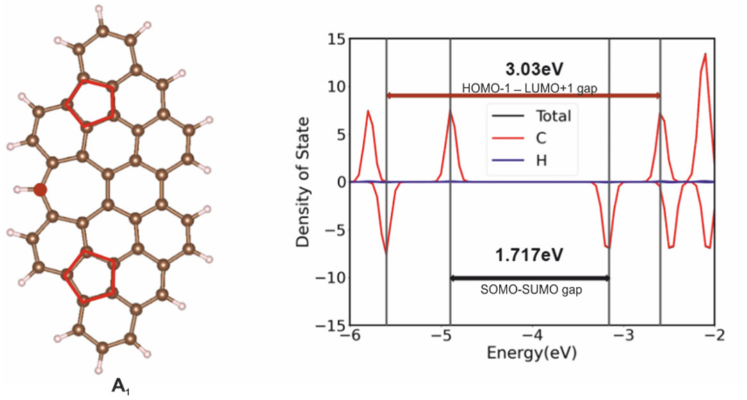

A_1

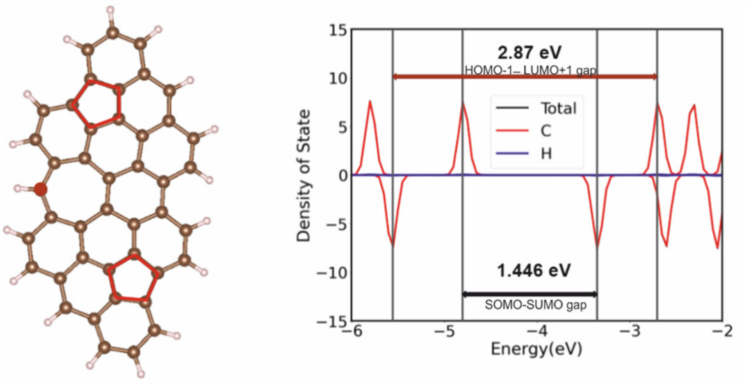

A_2

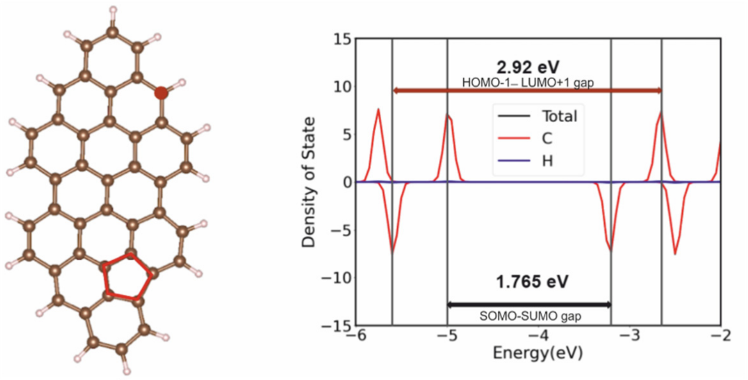

A_3

Figure S2. Scheme of calculated PDOS of the different open-shell nonbenzenoid NGs labeling the position of the frontier orbitals SOMO, SUMO, HOMO and LUMO.

3. Computational details

Total energy DFT calculations

Density functional theory (DFT) calculations were performed using FHI-AIMS code [2] using XC PBE0 functional [3,4] to describe the electronic properties of the gas-phase NGs. In all the calculations, we employed the light settings for the atomic basic sets. The atomic structures were thoroughly relaxed until the *Hellman*-Feynman forces were smaller than 10^{-3} eVÅ^{-1}. We employed PP-SPM code [4,5] to simulate constant-height d*I*/d*V* maps, shown on Figure 2d. CO-tip was represented by linear combination of s-like (15%) and p_x,p_y-like (85%) orbitals without tip relaxation at tip-sample distance 5 Å.

References

1. Zhang, F.-L.; Hong, K.; Li, T.-J.; Park, H.; Yu, J.-Q. Functionalization of C(Sp3)–H Bonds Using a Transient Directing Group. *Science* **2016**, *351*, 252–256, doi:10.1126/science.aad7893.

2. Blum, V.; Gehrke, R.; Hanke, F.; Havu, P.; Havu, V.; Ren, X.; Reuter, K.; Scheffler, M. Ab Initio Molecular Simulations with Numeric Atom-Centered Orbitals. *Comput. Phys. Commun.* **2009**, *180*, 2175–2196, doi:10.1016/j.cpc.2009.06.022.
3. Becke, A.D. A New Mixing of Hartree–Fock and Local Density-functional Theories. *J. Chem. Phys.* **1993**, *98*, 1372–1377, doi:10.1063/1.464304.
4. Hapala, P.; Kichin, G.; Wagner, C.; Tautz, F.S.; Temirov, R.; Jelínek, P. Mechanism of High-Resolution STM/AFM Imaging with Functionalized Tips. *Phys. Rev. B* **2014**, *90*, 085421, doi:10.1103/PhysRevB.90.085421.
5. Krejčí, O.; Hapala, P.; Ondráček, M.; Jelínek, P. Principles and Simulations of High-Resolution STM Imaging with a Flexible Tip Apex. *Phys. Rev. B* **2017**, *95*, 045407, doi:10.1103/PhysRevB.95.045407.
